# Robotic repair of a vesicovaginal fistula in an irradiated field using a dehydrated amniotic allograft as an interposition patch

**DOI:** 10.1007/s11701-015-0546-8

**Published:** 2015-12-11

**Authors:** David T. Price, Tina C. Price

**Affiliations:** East Texas Urology Specialists, 1111 West Frank Avenue, Suite 303, Lufkin, TX 75904 USA

**Keywords:** Vesicovaginal fistula, Pelvic irradiation, Robotic surgery, Amniotic graft, Wound healing

## Abstract

We report the case of a 66 year old female with a supratrigonal vesicovaginal fistula (VVF) that developed after undergoing radical hysterectomy, chemotherapy and pelvic radiation therapy for advanced cervical cancer. VVF repairs in an irradiated field are known to be complicated procedures with significant morbidity and a high rate of failure due to the effect of radiation. Amniotic membranes have been demonstrated to improve healing rates in difficult to heal wounds. To decrease morbidity a minimally invasive robotic procedure was performed and a dehydrated amniotic allograft patch was used to augment tissue healing. The VVF was repaired using the da Vinci Surgical System and the amniotic membrane was used as an interposition patch over the repair. There were no operative or postoperative complications and the patient was discharged home on postoperative day one. A cystogram performed 3 weeks postoperatively demonstrated a healed fistula. Follow-up at 5 months revealed no incontinence. This is the first reported case of a robotic VVF repair performed in an irradiated pelvis and the first use of an amniotic membrane allograft in the repair a VVF.

**Electronic supplementary material:**

The online version of this article (doi:10.1007/s11701-015-0546-8) contains supplementary material, which is available to authorized users.

## Introduction

Complex vesicovaginal fistulas (VVFs) represent a significant clinical challenge, and complex VVFs in an irradiated field are by nature some of the most difficult cases that urologists and gynecologists encounter. Treatment options for complex VVFs after radiation generally include either vaginal or abdominal open fistula repair utilizing various flaps or interposition grafts or urinary diversion with either percutaneous nephrostomy tubes, urinary conduit, or urterosigmoidostomy [1]. Unfortunately, the failure rate for primary surgical repair of complex VVFs after radiation is high, and this has led some to recommend urinary conduit as the preferred treatment option for patients with this clinical problem [2–4]. The high failure rate of VVF repairs in this patient population is primarily caused by the destructive effects of radiation on the microvasculature which results in poor wound healing.

There are limited reports of using laparoscopic and robotic techniques to repair complex VVFs in general, and there are no reported cases of robotic VVF repair in an irradiated field [5–7]. In fact, there is only one single case of laparoscopic VVF repair in an irradiated field [8]. In this manuscript, we report the first successful robotic VVF repair in an irradiated field utilizing a dehydrated amniotic membrane as an interposition graft to assist in wound healing.

## Case report

A 66 year old female presented with total uncontrolled urinary incontinence for over 6 months. Past medical history was notable for locally advanced cervical cancer treated by radical hysterectomy, chemotherapy and 4500 cGy pelvic radiation. She was diagnosed with a 1 cm solitary supratrigonal VVF by clinical exam and cystoscopy. Treatment options were discussed and she adamantly opposed undergoing a urinary diversion. She elected and consented to undergo a robotic repair of the VVF using an amniotic membrane allograft.

**Fig. 1:**
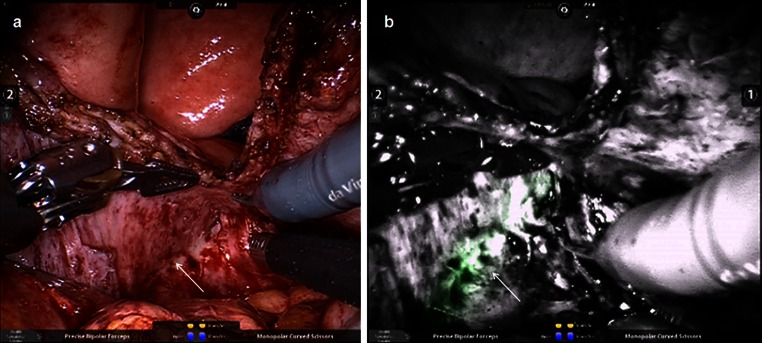
Intraoperative photograph of illuminated catheter (Cook Medical) used to identify the ureter during dissection. It is visualized with: **a** normal light and **b** Firefly Fluorescence Endoscopy (Intuitive Surgical, Inc.). *Arrows* demonstrate location of ureter

**Fig. 2:**
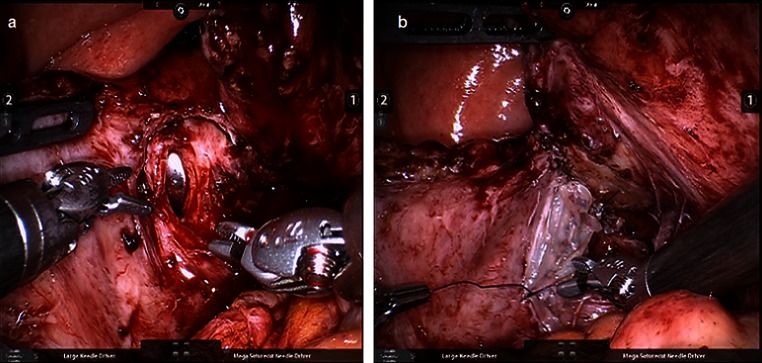
Intraoperative photographs of: **a** open bladder with EEA sizer exposing the vesicovaginal fistula after excising fibrotic edges and **b** rehydrated amniotic membrane patch sutured in place over the surface of vaginal side of the fistula after performing a two layer closure of the vaginal opening

The operative procedure was performed in lithotomy and exaggerated Trendelenburg position as one would position a patient for a robotic prostatectomy. Illuminating ureteral catheters (Cook Medical, Bloomington, IN) were placed to assist with ureteral identification intraoperatively, and a standard flexible ureteral catheter was placed thru the cystoscopy and passed through the fistula tract. An endopath veress needle (Ethicon Endo Surgery, Cincinnati, OH) was utilized to establish pneumoperitoneum. A standard fan array utilizing the fourth arm on the patient’s left was utilized for port placement. The patient had extensive intra-abdominal adhesions requiring prolonged lysis of adhesions between the bowel, omentum, and pelvic structures. The da Vinci SI Surgical Robot (Intuitive Surgical, Inc., Sunnyvale, CA) was positioned between the legs and Firefly Fluorescence Endoscopy (Intuitive Surgical, Inc.) was utilized intermittently for intraoperative visualization of the ureters while the fistula tract was dissected (Fig. 1). The fistula was approached using a modification of the technique described by O’Conor and Sokol [9]. The fistula was dissected extravesically as far as possible then a vertical cystotomy was performed. The fistula tract was completely excised and sent for pathology. The bladder and vaginal edges were then mobilized and trimmed so that a repair could be made with no tension. The vaginal edges of the fistula were exposed using an end-to-end anastomosis (EEA) sizer placed in the vagina (Fig. 2a). The vaginal and bladder fistula defects were then closed in two layers using interrupted 4-0 polydixanone (PDS, Ethicon). Amniofix (MiMedx, Marietta, GA), a dehydrated amniotic membrane allograft, was then inserted through the assistant port rolled around a grasper. The membrane was placed over the repaired vaginal defect and allowed to hydrate and was then carefully sutured in place using 4-0 polydixanone (Fig. 2b). The bladder cystotomy was then closed using two layers of running 3-0 polyglactin (Vicryl, Ethicon). The bladder repair was tested using bladder distension with saline. A 19 French Blake drain (Ethicon) was placed in the pelvis.

The patient underwent an uncomplicated surgical procedure with the fistula identified and excised in standard fashion. Pathological evaluation of the fistula was benign. Total anesthesia time including cystoscopy with catheter placement, repositioning patient, lysis of adhesions, and the robotic procedure was 305 min. The robotic operative time was approximately 240 min and the blood loss was 50 cc. There were no intraoperative or postoperative complications. She was discharged home on postoperative day one with a Foley catheter and Blake drain. On postoperative day three, the drain output was minimal with a normal creatinine level and the drain was removed. She was continued on oral suppressive antibiotics while she had a Foley catheter. A cystogram performed at 3 weeks postoperatively demonstrated resolution of the fistula and the catheter was removed. Follow-up at 5 months revealed no incontinence or recurrence of the fistula.

## Discussion

Open surgical approaches have been the preferred approach for managing complex irradiated VVFs for over 50 years; however, they can be associated with significant morbidity and can result in prolonged hospitalization [10]. While the benefits of minimally invasive surgical techniques in decreasing morbidity and hospital length of stay are well recognized, there have been few attempts to utilize these techniques for the repair of these types of complex fistulas. The laparoscopic application of the O’Conor technique has been applied to a single patient with a complex radiated VVF with a successful outcome [8]. However, the complex technical skills required to perform this procedure will more than likely prevent its use on a widespread basis.

In this case, the fistula was approached using a robotic modification of the O’Conor approach [9]. The da Vinci surgical robot provided improved visualization and technical advantages which allowed the procedure to be performed successfully; however, the difficulty level of this case should not be discounted. The approach was challenging, even for an experienced robotic surgeon, so it should be considered a complex robotic case that should not be attempted until gaining experience on less challenging cases. Nevertheless, the approach is feasible, and as demonstrated by this case it can be performed with minimal morbidity.

Unfortunately, one would expect a high rate of fistula recurrence to plague even well performed minimally invasive VVF repairs in the irradiated field as they have for decades with open procedures [2–4]. This is largely because the irradiated pelvis is a hostile and unforgiving environment. Factors necessary for normal wound healing in the irradiated pelvis are lacking, and it is for this reason that normally a well vascularized pedicle of either muscle, fat, peritoneum or omentum is used as an interposition graft [2–4]. The vascularized pedicle serves as a barrier and theoretically introduces vascularity to the area. However, in one reported series of patients undergoing repair of recurrent VVFs, where an omental interposition graft had been used, the authors noted a lack of neovascularity in the area of the primary fistula repair and in many cases the omental graft itself was missing [7]. This raises some question as to how effective the standard omental graft actually functions in this setting, and this could at least partially explain some of the poor outcomes seen with open surgical repair of irradiated VVFs.

Human amniotic membranes have been used for decades as biological dressings on a variety of difficult to heal wounds including those in irradiated tissues [11, 12]. Amniotic membranes act as a biological barrier, function as a scaffold for tissue ingrowth, and release peptide growth factors required for tissue growth, collagen deposition, and angiogenesis [13, 14]. In vitro studies have documented that extracts prepared from human amniotic membrane allograft contain peptide growth factors that stimulate the proliferation and migration of human derived vascular endothelial cells in cell culture. Furthermore, in murine experiments, subcutaneously implanted allografts demonstrated a dynamic neovascularization process with a steady increase in blood vessel density over a 4 week period [14]. Dehydrated amniotic membranes are now available commercially, and they have been demonstrated to significantly improve the rate of wound healing in compromised tissues such as diabetic foot ulcers [15, 16]. In one prospective randomized controlled study where dehydrated amniotic membranes were compared to traditional care of diabetic foot ulcers the rate of healing was significantly better for those treated with dehydrated amniotic membranes compared to traditional care 92 versus 8 %, respectively [15]. Since diabetic foot ulcers demonstrate some of the same findings present in the irradiated field with decreased wound healing secondary to microvascular damage, we hypothesized that the membrane would serve as a physical barrier between the repaired bladder and vaginal sides of the repaired fistula in the irradiated pelvis and the biological properties of the membrane might improve the chances for a successful outcome.

Amniofix (MiMedx, Inc), the allograft used in this particular case, has been extensively studied and demonstrated to release a variety of growth factors involved in wound healing. It also provides a barrier and acts as a tissue matrix that promotes fibroblast ingrowth and angiogenesis [13–16]. One problem encountered with using the dehydrated membrane was its fragility and it must be handled with care to avoid tearing. Nevertheless, it was possible to use the membrane as a patch, and it appears to have functioned well in this case. Another potential problem with the dehydrated allograft is the duration of the angiogenic effect in vivo remains unknown and patients with radiation fistulas remain at risk for recurrent fistulas for years. We would have preferred to add an additional vascularized patch of either omentum or peritoneum in addition to the amniotic membrane, but this was not possible in this case.

Since the available clinical evidence demonstrate that amniotic membranes improve healing rates for difficult to heal wounds including those in radiated tissues, it would be reasonable to speculate that these types of membranes might play an important role in the support of healing complex fistula repairs performed in the irradiated pelvis. Likewise, the application of the technique of using amniotic membranes as a bolster may be useful following colon resection on irradiated bowel where anastomotic leak rates are high. While this case provides supplemental support for this hypothesis, further clinical experience and additional trials utilizing these membranes would be necessary; however, given the high rate of fistula recurrences in these patient populations such studies would seem both reasonable and warranted.

## Electronic supplementary material

Supplementary material 1 (MP4 53677 kb)
